# Optimization strategies of in-tube extraction (ITEX) methods

**DOI:** 10.1007/s00216-015-8854-4

**Published:** 2015-06-30

**Authors:** Jens Laaks, Maik A. Jochmann, Beat Schilling, Torsten C. Schmidt

**Affiliations:** Instrumental Analytical Chemistry, University Duisburg-Essen, Universitätsstrasse 5, 45141 Essen, Germany; BGB Analytik AG, Lettenstrasse 97, 8134 Adliswil, Switzerland

**Keywords:** In-tube extraction, ITEX, ITEX DHS, Method development, Parameter optimization

## Abstract

Microextraction techniques, especially dynamic techniques like in-tube extraction (ITEX), can require an extensive method optimization procedure. This work summarizes the experiences from several methods and gives recommendations for the setting of proper extraction conditions to minimize experimental effort. Therefore, the governing parameters of the extraction and injection stages are discussed. This includes the relative extraction efficiencies of 11 kinds of sorbent tubes, either commercially available or custom made, regarding 53 analytes from different classes of compounds. They cover aromatics, heterocyclic aromatics, halogenated hydrocarbons, fuel oxygenates, alcohols, esters, and aldehydes. The number of extraction strokes and the corresponding extraction flow, also in dependence of the expected analyte concentrations, are discussed as well as the interactions between sample and extraction phase temperature. The injection parameters cover two different injection methods. The first is intended for the analysis of highly volatile analytes and the second either for the analysis of lower volatile analytes or when the analytes can be re-focused by a cold trap. The desorption volume, the desorption temperature, and the desorption flow are compared, together with the suitability of both methods for analytes of varying volatilities. The results are summarized in a flow chart, which can be used to select favorable starting conditions for further method optimization.

**Electronic supplementary material:**

The online version of this article (doi:10.1007/s00216-015-8854-4) contains supplementary material, which is available to authorized users.

## Introduction

Method development for microextraction techniques can be a very time-consuming task, because a multitude of different parameters influence the efficiency of extraction. Even in the simplest system, where only a coated fiber (solid-phase microextraction or SPME) is immersed in a liquid sample, the extraction can be influenced by (i) the choice of the polymeric coating, (ii) the extraction time together with (iii) shaking or stirring, (iv) the extraction temperature, (v) the pH for ionizable compounds, (vi) the ionic strength, and (vii) the presence of organic solvents or matrix compounds such as humic substances [1]. Dynamic microextraction techniques, where the sample is actively passed over the sorbent material or through a sorbent bed, are more complex and thus have even more parameters to optimize during the steps of the extraction and thermal desorption procedure, e.g., the volume and the corresponding flows that are applied during extraction and desorption [2–4].

**Fig. 1:**
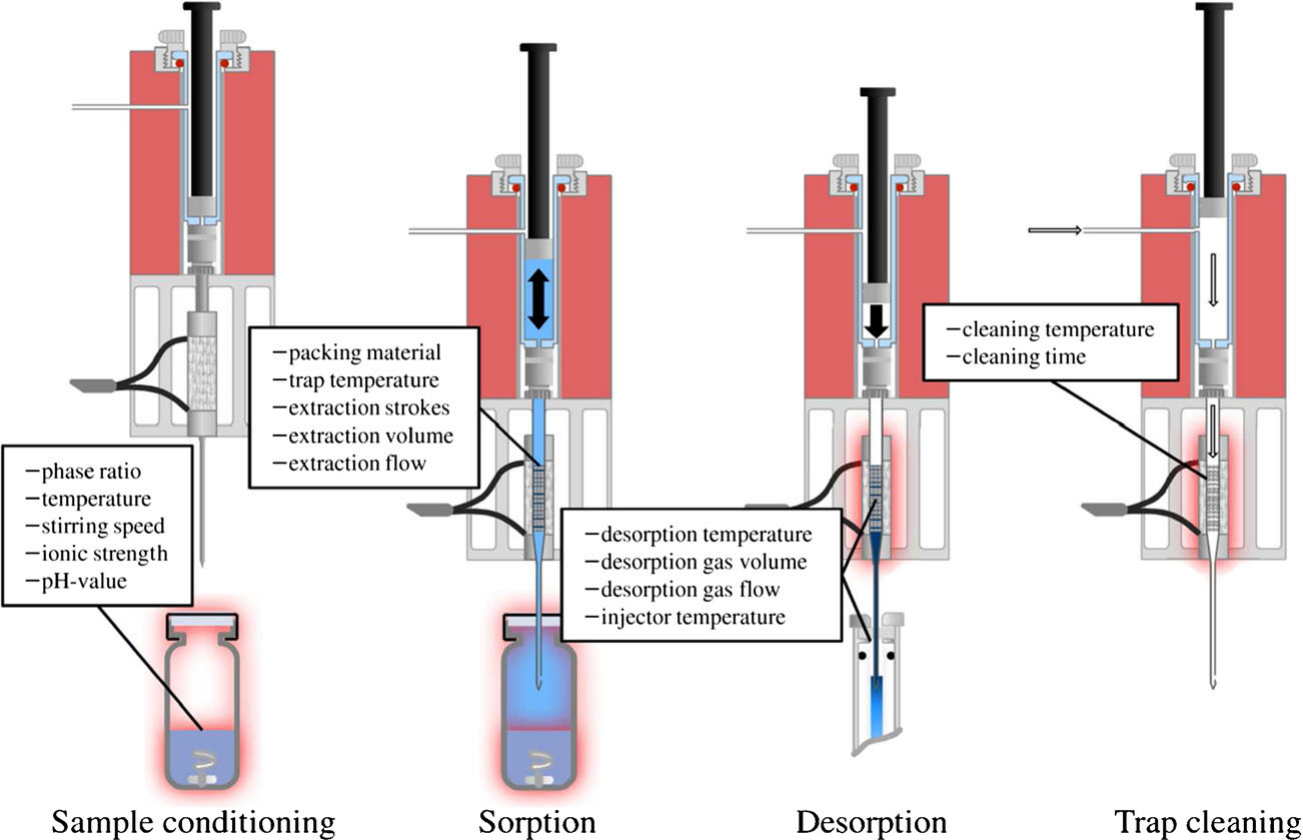
Stages of the ITEX procedure with the corresponding parameters for optimization, adapted from [4]

In-tube extraction (ITEX) is a fully automated microextraction technique for CTC PAL series autosamplers and uses a gastight syringe to pump the sample headspace repeatedly through an attached tube, filled with a sorbent material for analyte enrichment. The syringe, as well as the sorbent tube, is enclosed by an electric heater to avoid sample condensation in the syringe and to facilitate thermal desorption to the inlet system of the gas chromatograph, respectively. The syringe also features a side-port hole in the glass body, which allows the flushing of the syringe and the sorbent tube with a pure, inert gas for trap conditioning to avoid carryover between analyses. The four stages of the ITEX procedure (sample conditioning, analyte extraction/sorption, desorption/injection, and trap conditioning), together with the main parameters governing the performance of each stage, are depicted in Fig. 1 [4].

The aim of this work is to summarize the experiences gained in the ITEX method development and to present a guideline that allows future user to minimize the number of experiments, which are required to find the appropriate parameters for their analytical task.

## Experimental

### Target compounds

The target compounds used in the developed methods can be sorted into two categories: volatile organic compounds (VOCs) as water contaminants and aroma compounds in food matrices. The VOCs are comprised of halogenated hydrocarbons, BTEX compounds (benzene, toluene, ethylbenzene, xylenes), and gasoline oxygenates [ethyl *tert*-butyl ether (ETBE), methyl *tert*-butyl ether (MTBE), and *tert*-amyl methyl ether (TAME)]. The volatile compounds include several alcohols, aldehydes, esters, terpenes, and 2,3-butanedione, pyridine, methylpyrazine, and 2-furanmethanol. A complete list, together with the sample matrix and the used sorbent material, is given in Table 1.

**Table 1:** Analyzed target compounds with corresponding sample phase and sorbent material

VOCs [4]	Aroma compounds	
Sample matrix		
Water	Beer	Coffee powder
Sorbent material		
Tenax GR/Carbosieve SIII	Tenax TA, PDMS	PDMS
Target compounds		
Vinyl chloride	1-Propanol	Acetaldehyde
Dichloromethane	2-Propanol	Propanal
Chloroform	2-Methylpropanol	2-Methylpropanal
1,2-Dichloroethane	1-Butanol	2-Methylbutanal
Trichloroethylene	2-Butanol	3-Methylbutanal
Bromodichloromethane	*tert*-Butanol	2,3-Butanedione
Tetrachloroethylene	2-Methylbutanol	Pyridine
Dibromochloromethane	3-Methylbutanol	Methylpyrazine
Bromoform	1-Pentanol	2-Furanmethanol
MTBE	3-Pentanol	
ETBE	3-Methylpentanol	
TAME	1-Hexanol	
Benzene	2-Ethylhexanol	
Toluene	Ethyl acetate	
Ethylbenzene	Ethyl butanoate	
*p*-Xylene	Ethyl 3-methylbutanoate	
*o*-Xylene	3-Methylbutyl acetate	
1,4-Dioxane	Ethyl hexanoate	
2-Methylisoborneol	Ethyl octanoate	
Geosmin	Ethyl decanoate	
	Diethyl succinate	
	2-Phenethyl acetate	
	Geraniol	
	Linalool	

### Instrumentation

The experiments were performed on two instruments. The first instrument was a Thermo Trace GC Ultra (S+H Analytik, Mönchengladbach, Germany), equipped with a CTC Combi PAL autosampler with ITEX-2 option (Axel Semrau, Sprockhövel, Germany) and a Single Magnet Mixer (SMM) (Chromtech, Idstein, Germany); the autosampler was modified with a small electric fan for faster cooling of the ITEX trap. The GC featured a split/splitless injector (SSL) and an Atas GL Optic 3 programmable temperature vaporizer with a nitrogen-cooled cold trap for on-column focusing (Axel Semrau). On-column focusing was performed on a deactivated, uncoated 0.53-mm-inner diameter (i.d.) fused silica capillary with a length of about 1 m (BGB Analytik AG, Boeckten, Switzerland). A Rtx-VMS column (medium polar, proprietary modified phase) with a length of 60 m, i.d. of 0.32 mm, and film thickness of 1.8 μm (Restek GmbH, Bad Homburg, Germany) was used for the separation of VOCs, and a Stabilwax-DA fused silica capillary column [cross bonded carbowax (polyethylene glycol (PEG))] with a length of 60 m, i.d. of 0.32 mm, and film thickness of 1 μm (Restek GmbH) was used for the separation of aroma compounds. The GC was coupled to a Thermo DSQ II single-quadrupole mass spectrometer (S+H Analytik, Mönchengladbach, Germany), operated in EI mode for analyte detection.

The second instrument was a ThermoQuest Trace GC (ThermoQuest GmbH, Egelsbach, Germany) outfitted with a CTC PAL Combi-xt with ITEX-2 option (Axel Semrau) and a SMM. The GC had a split/splitless injector, an Optima 5 MS (5 % diphenyl-95 % dimethylpolysiloxane) fused silica chromatographic column with a length of 30 m, i.d. of 0.25 mm, and film thickness of 0.25 μm (Macherey-Nagel GmbH & Co. KG, Düren, Germany) was installed for analyte separation, and a Finnigan Polaris Q (ThermoQuest GmbH) external source ion trap mass spectrometer was connected as a detector in EI mode.

If not stated otherwise, 10 mL of each standard or sample solution was transferred into a 20-mL amber headspace vial (BGB Analytik AG), containing an 8 × 3 mm PTFE-laminated magnetic stir bar (VWR International GmbH, Darmstadt, Germany), which were closed by magnetic screw caps with rubber/PTFE septa (BGB Analytik AG).

### Sorbent materials

The applied sorbents are mostly standard materials, which are also used in desorption tubes for gas analysis, in purge and trap instruments or as stationary phase in packed GC columns. Carbopack C (CC), Carboxen 1000 (C1000), Carbosieve SIII (CSIII), Tenax TA (TTA), and Tenax GR (TGR) are commercially available as single- and also as multi-sorbent ITEX traps, while HayeSep D (HSD), multi-walled carbon nanotubes (MWCNTs) (Baytubes C 150 HP; Bayer Material Science, Leverkusen, Germany), polydimethylsiloxane (PDMS), and Carbowax 20M (PEG with a molecular weight of 20,000) are custom-prepared taps, which were, to our knowledge, first used here. The properties of the applied sorbent materials are given in Table 2.

**Table 2:** Applied sorbent materials and, if not stated otherwise, properties by manufacturer

Sorbent	Sorbent type	Specific surface area (m^2^ g^−1^)	Temperature limit (°C)	Water affinity	Typical applications
Carbopack C	Graphitized carbon black	10	500	Relatively low	Low to medium boilers (C12–C20)
Carboxen 1000	Carbon molecular sieve	1200	225	Moderate	Permanent gases, volatiles (C2–C5)
Carbosieve SIII	Carbon molecular sieve	975	400	Moderate	Volatile organics (C2–C5)
Tenax GR	70 % porous organic polymer/30 % graphitized carbon	24	350	Low	Volatiles, flavors
Tenax TA	Porous organic polymer	35	350	Low	Volatiles and semi-volatiles (C7–C26)
HayeSep D	Porous organic polymer	795	290	Low	Volatiles (C1–C6)
MWCNT	Multi-walled carbon nanotubes	211^a^	n.a.	n.a.	n.a.
PDMS	Silicone rubber	Absorbent	250	Low	Nonpolar volatiles, semi-volatiles
Carbowax 20M	Polyethylene glycol	Absorbent	225	High	Polar semi-volatiles

*n.a.* not available

^a^BET measurement

## Results and discussion

The effects of the essential parameters of the ITEX procedure will be discussed here with detailed examples; they include the selection of the sorbent material and the extraction and injection parameters but also ways to shorten the analysis time by modifications of both, the ITEX hardware and the macros of the control software. The initial step of sample conditioning will not be discussed here, because it is basically the same as for other headspace techniques, which can be found in the literature [5].

### Sorbent selection

#### Theoretical considerations

The first step in ITEX method development should be the selection of a suitable sorbent for the analytical task. One way to achieve this is to compare the extraction efficiency of all available sorbent materials for all target analytes, like it has been performed here for the sake of completeness. Another option is to save time and limit the number of possible extraction phases, based on the target compounds and sample characteristics. To that end, it is important to check for known unintended interactions between analytes and sorbents. For example: (i) activated carbon possesses several functional groups like hydroxyl, carbonyl, and carboxylic functions where polar analytes like alcohols might be adsorbed, irreversible by thermodesorption, through hydrogen bonds [6]; (ii) the surface of carbon-based adsorbents can be activated during conditioning (even in a stream of inert gas) and then cause analyte loss by transformation reactions, especially for alcohols and carbonyl compounds [7–9]; and (iii) Tenax is known to release aldehydes (e.g., benzaldehyde) and ketones during thermodesorption, which can obscure the determination of these compounds [10, 11, 7]; on the other hand, the degradation products of PDMS can easily be identified using mass selective detectors and are usually unproblematic [12, 13].

The sorbent materials suitable for ITEX can be separated into two classes: adsorbents and absorbents. Adsorbents rely on surface interactions of the sorbent material with analyte molecules, while in absorption, the analyte molecules are solvated in the extraction phase like in an organic solvent. Absorptive interactions are weaker than adsorption on active surfaces, which makes the trapping of highly volatile analytes difficult but also allows lower desorption temperatures and shorter desorption times, which minimizes the degradation of unstable analytes [7, 13]. Because the available active sites on the adsorbent surface are limited, problems in quantitative analysis can occur, when the analyte mass is high (either by too high concentration or too large sample amount), due to competition or displacement effects, while the equilibrium conditions of absorbents do not vary until the extracted amount is large enough (a few percent of the sorbent mass) to modify the properties of the sorbent phase [14]. This makes adsorbent materials ideal for trace/ultra-trace analysis of samples with little matrix interferences and for samples where all analytes are in a similar concentration range [4], so that a saturation of the sorbent can be excluded. Absorbents in contrast are best used when the concentration range of the analytes is wide or high concentrated matrix components could saturate an adsorbent, for example like ethanol often does in the aroma analysis of alcoholic beverages [15].

#### Exemplary extraction efficiencies

**Fig. 2:**
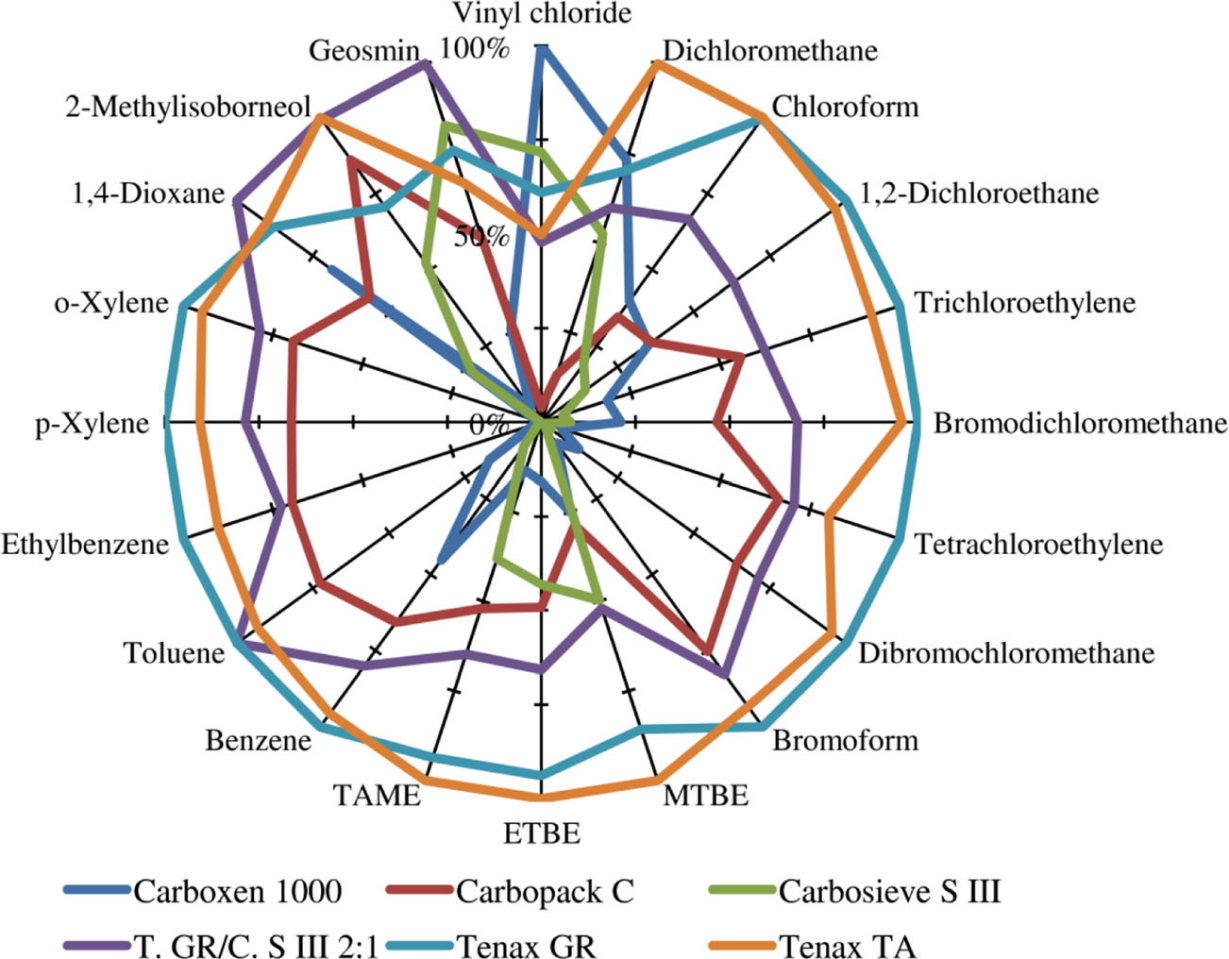
Relative extraction yields of six tested standard sorbent traps for the analysis of VOCs, and the result for each compound was normalized to the most efficient sorbent (data taken from [4])

In the following, the relative extraction efficiencies of commercially available, but also of custom-prepared ITEX traps, obtained for the analytes listed in Table 1 will be discussed. To that end, for each compound, the sorbent with the highest resulting peak area was used as a reference to normalize the peak areas of the other traps. The results for the analysis of VOCs are shown in Fig. 2. The overall best extraction yield was achieved with TGR, which was the most efficient for 11 compounds, followed by TTA which had the highest yield for 6 compounds and very similar results as TGR for most other analytes, while C1000 was the most efficient for vinyl chloride, and the mixed TGR and CSIII trap was good for 1,4-dioxane, 2-methylisoborneol, and geosmin.

The relative extraction yields of aroma compounds from beer analysis have been split into two diagrams, because of the higher number of evaluated sorbents, but both corresponding diagrams were normalized to the same scale. The standard sorbents, also used for the VOC analysis, are shown in Electronic Supplementary Material (ESM) Fig. S1, and the custom-filled traps, first applied in this project, are shown in ESM Fig. S2. While the sorbents with the highest yields were TTA for alcohols and HSD for longer-chain esters and terpenes, a lower, but more balanced performance for all compounds could be observed for the PDMS-containing traps. The average extraction yield of the C1000 and CSIII traps was quite low, except for few compounds like ethyl acetate and propanol.

The extraction yields of major coffee aroma compounds are shown in ESM Figs. S3 and S4. Good results for the extraction of acetaldehyde could be obtained with CSIII and C1000, while they were not as well suited for the other analytes. PDMS and MWCNT traps showed a low applicability for these compounds compared to TGR, TTA, and HSD, which are optimal for their extraction.

### ITEX extraction

#### Sorbent and sample temperature

**Fig. 3:**
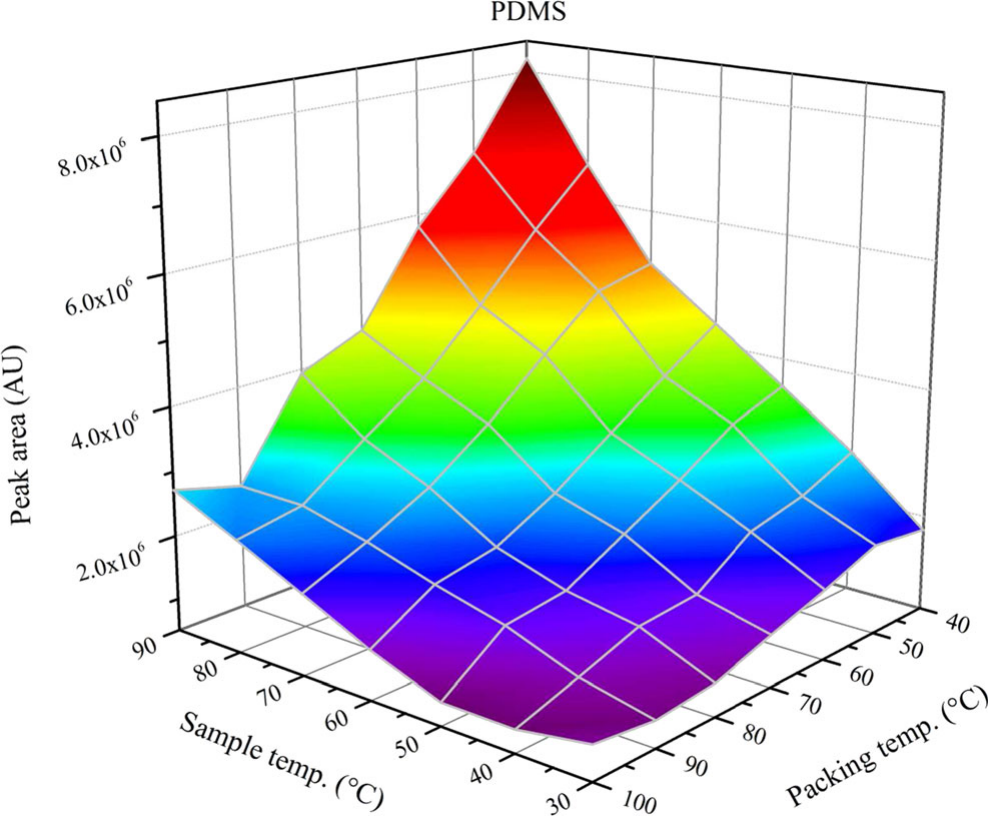
Influence of sample and packing temperature on the resulting peak area of toluene for PDMS

**Fig. 4:**
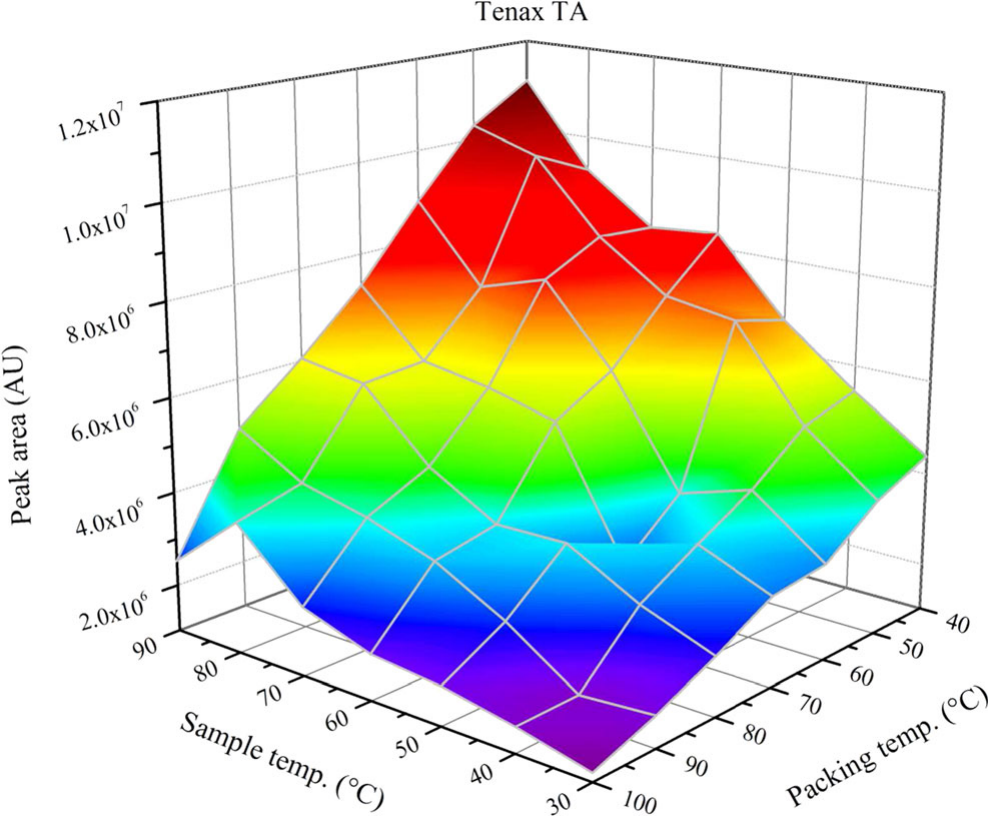
Influence of sample and packing temperature on the resulting peak area of toluene for Tenax TA

Another parameter that can be set without much experimental effort is the trap temperature; it should be set to the lowest value that can be obtained in the laboratory, because analyte sorption to the trap material is typically an exothermic process [16, 17]. On the other hand, the air-water partitioning coefficient increases with higher sample temperatures, which results in a competition between both effects, when the sorbent phase is inserted directly into the sample vial like it is the case with techniques like SPME or SPDE. In this case, it is an advantage of the ITEX device that the sorbent material is placed in a tube outside the heated sample vial and that the trap temperature can be controlled independently from the conditioning temperature of the sample. However, when the temperature difference between the sample vial and the trap becomes too large, problems with condensation of water on the sorbent material can arise, depending on the sorbent material. The influence of the sample and sorbent temperature on the extraction efficiency of toluene from water with four different sorbent materials, with increasing water affinity, is shown in Figs. 3 and 4 and ESM Figs. S5 and S6. For all investigated sorbents, the peak area increases in the direction of rising sample temperatures and decreasing packing temperatures and the highest peak area tends to be reached, when the highest sample and the lowest packing temperatures are applied. However, it is also visible that for the sorbents with higher water affinity, i.e., CSIII or PEG, the structure of the plotted surface shows discontinuous behavior at those points, where the sample temperature is higher than the packing temperature, which is most likely caused by water condensing on the sorbent surface, influencing the precision and accuracy of the measurement.

#### Extraction flow and extraction strokes

While the extraction flow through the trap and the number of performed extraction strokes are the defining factors for the extracted amount and necessary extraction time in analytical applications, they are mostly optimized to the maximum extraction yield, individually [3, 18]. Although most authors used a method with a high extraction flow and a large number of extraction strokes [3, 19, 20, 18, 15], the measurement of different combinations of extraction strokes and flows has so far only been reported, recently [21, 4]. Therefore, after discussing both individual parameters, special emphasis will be laid on the interaction of both parameters to achieve an optimum extraction yield in a predefined extraction time, for instance in parallel to the GC oven runtime.

**Fig. 5:**
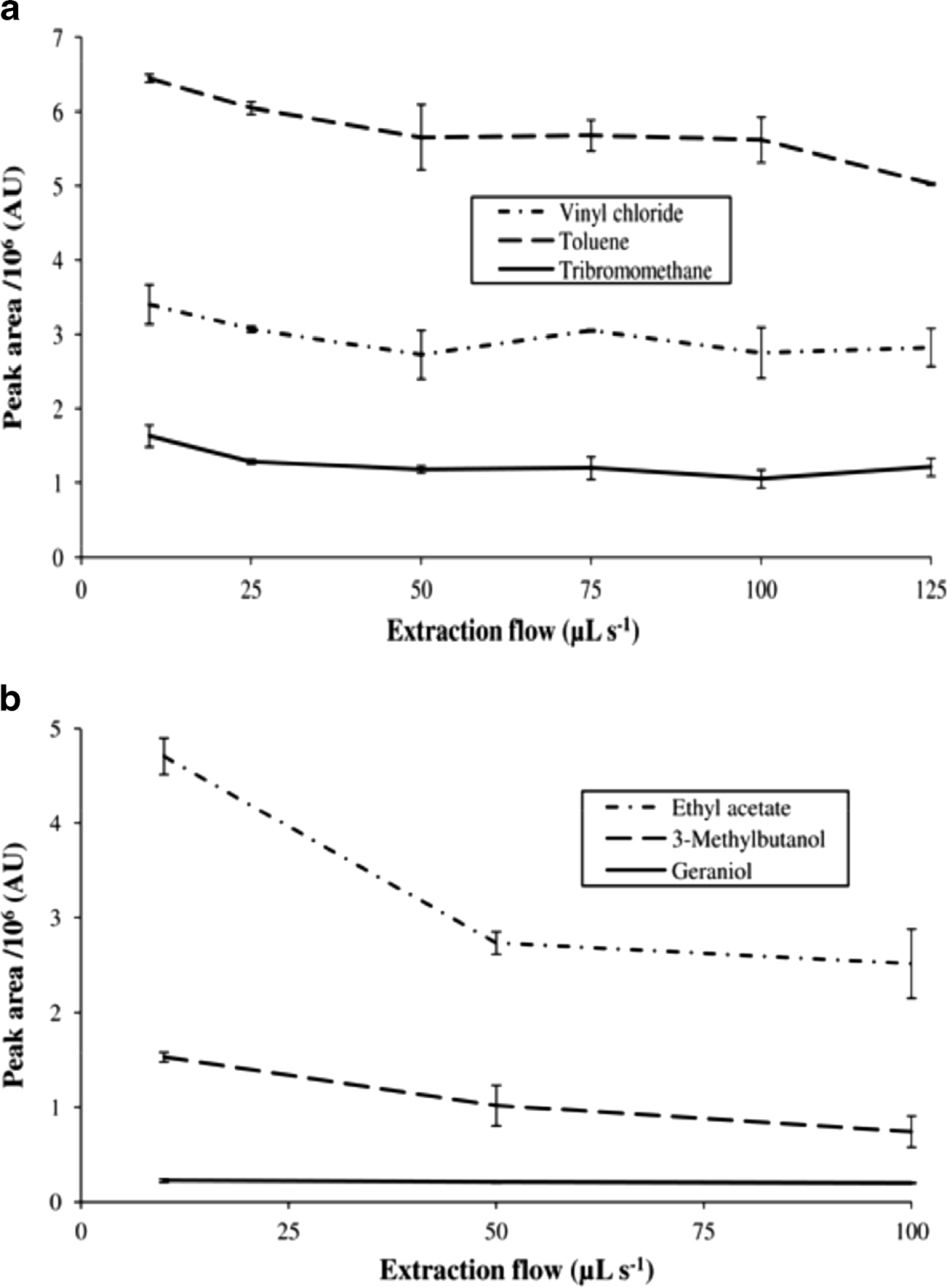
(**a**) Influence of the extraction flow on the obtained peak areas of vinyl chloride, toluene, and tribromomethane with a TGR/CSIII trap using 10 extraction strokes, from a 23-compound standard mixture with a concentration of 1 mg L^−1^ per compound. (**b**) Influence of the extraction flow on the obtained peak areas of ethyl acetate, 3-methylbutanol, and geraniol with a TTA trap using 75 extraction strokes from a 24-component standard mixture with a concentration of 5 μg L^−1^ per compound

Figure 5a, b shows the influence of the extraction flow on the extraction yield of six analytes from different compound classes, using a TGR/CSIII trap and a TTA trap. As a general trend, it could be observed that the extraction yield decreased towards higher extraction flows and that the effect was stronger at lower analyte concentrations. The largest influence was observed for ethyl acetate, where the extraction yield at an extraction flow of 10 μL s^−1^ was almost twice as high as at 100 μL s^−1^, whereas no significant influence on the extraction yield could be observed for geraniol and vinyl chloride. Jochmann et al. suggested diffusion into the sorbent pores to be the rate-limiting effect at higher extraction flows [3], as it can also be observed by increasing plate heights in gas chromatography [22]. The lesser retention of analytes would also result in a lower breakthrough volume; however, this can be neglected as ITEX is a closed sampling system. Furthermore, the extraction flow was the parameter with the least influence on extraction yield, when it was compared to the extraction temperature and the number of extraction strokes [21], which allows more flexibility to achieve time efficient analyte enrichment.

The peak areas, obtained from the extraction of the headspace of a toluene solution with 1 mg L^−1^, using a TTA trap with varying numbers of extraction strokes, are presented in Fig. 7. The development of the peak areas can be separated in two ranges; the increase was linear from the beginning up to 10 extraction strokes and then changed to a logarithmic trend until the upper limit of 100 strokes. At the start of the extraction process, the pre-conditioned sorbent material was not loaded and all analytes, which were pumped through the sorbent bed, were trapped; therefore, the resulting peak areas were proportional to the sampled volume, which is defined by the number of extraction strokes [23]. The loading on the trap increased with the sampled volume until the analytes, which were transported through the sorbent bed with each extraction stroke, could not be trapped completely, anymore. From this point on, the increase of peak areas changed to the logarithmic trend and, in an open sampling system, this would result in analyte loss [24], but as ITEX is a closed system, the analyte fraction that was not adsorbed would be re-injected to the sample vial. The sampling volume with a linear increase depends on the distribution constant between the analyte and the sorbent, the amount of used sorbent, and the analyte concentration [23]; while it extended up to 20 extraction strokes for the low volatile geosmin, no linear trend could be observed for the very volatile vinyl chloride in both cases under the same conditions as for toluene [4]. The following logarithmic trend was also observed in other experiments that were performed up to 200 extraction strokes (data not shown), and which data are not shown here, because they were only performed with single determinations due to the long extraction times. In this way, the response per extraction stroke could easily be predicted by just a few measurements only. However, this simple relation is only valid for low analyte masses, because the limited sorption sites of adsorbent materials lead to saturation effects, when samples with higher concentrations or mixtures containing several compounds are analyzed. While toluene alone did not reach equilibrium in more than 100 extraction strokes, it only took 40 extraction strokes to reach a steady state in a mixture containing 23 compounds. When the extraction is continued beyond this point, analytes with a low affinity to the sorbent material can be displaced by stronger retained analytes. This will show as a nonlinear behavior in the calibration functions at the higher concentrated mixed standard solutions. Thus, the number of extraction strokes can be used to tune the sensitivity of the method to the expected concentration level of the samples; a high number of extraction strokes should be applied for trace analysis in the nanograms per liter to micrograms per liter range, while for higher concentrated samples in the milligrams per liter range, a lower number might be more suitable.

**Fig. 6:**
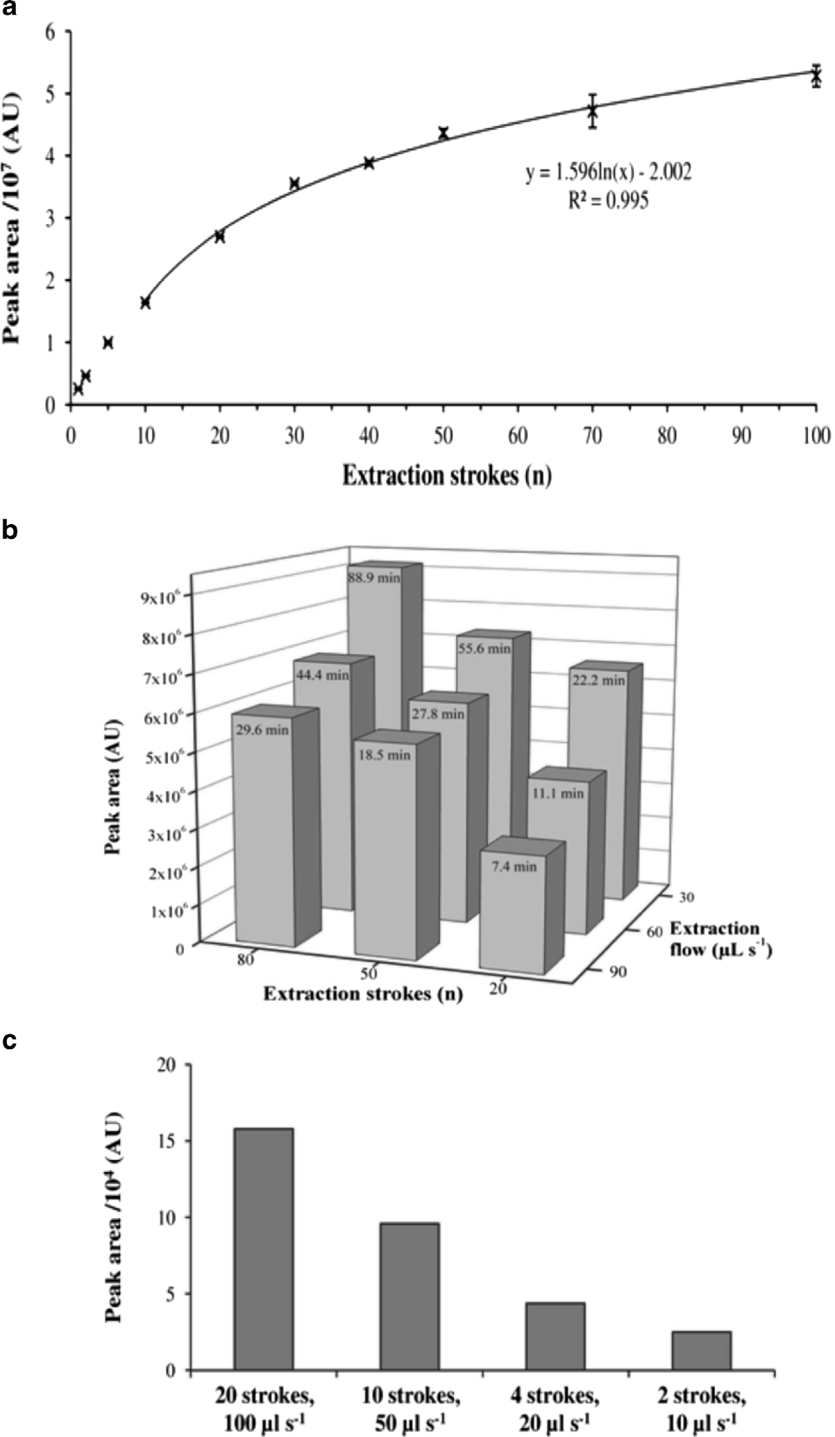
(**a**) Influence of the number of extraction cycles on the extraction yield of toluene. (**b**) Effects of combinations of varying numbers of extraction strokes and extraction flows on the extraction yield of toluene on MWCNTs, together with the resulting extraction time of each combination (unpublished data from [21]). (**c**) Toluene extraction yield of a TTA trap for different combinations of extraction strokes and flows, resulting in a constant extraction time of 6.7 min

The peak areas of nine combinations of extraction flows and extraction strokes are presented in Fig. 6b, accompanied by the resulting extraction time for each combination. As expected, the peak areas increased towards lower extraction flows and also towards more extraction strokes. The results for most combinations, apart from 80 extraction strokes with 30 μL s^−1^ or 20 extraction strokes with 90 and 60 μL s^−1^, were quite similar, but the necessary extraction times varied from 18.5 to 55.6 min. Thus, the calculation of the extraction efficiency, as a peak area obtained per second of extraction, can be a good way to identify the most suitable extraction parameters, which is given in ESM Table S1.

The highest extraction efficiencies were achieved at 20 extraction strokes with 60 and 90 μL s^−1^, because they mainly cover the linear part of the extraction profile (see Fig. 6a), however with small peak areas. The most efficient extraction of the combinations with similar peak areas was achieved with 50 strokes at 90 μL s^−1^ and 20 strokes at 30 μL s^−1^ with 5.0 and 4.9 kAU s^−1^, respectively. Four combinations which result in a constant extraction time are compared in Fig. 6c; here, the higher number of fast extraction strokes by far outperforms the lower extraction flows. However, these differences might diminish when longer extraction times are chosen, as described above.

### ITEX and injection to GC

The most suitable parameters for ITEX needle injections depend on the volatility of the analytes of interest and the technical configuration of the GC. Based on these preconditions, two general cases can be distinguished: (i) the analytes cannot be re-focused on the head of the analytical column or (ii) the analytes can be re-focused by either a low oven temperature or a cryogenic cooling trap. The consequences will be discussed in the following section.

#### Injection without analyte focusing

There are two injection methods for the ITEX technique. The first (hence denoted as ITEX_inj) aspirates the defined injection volume of a desorption gas and then starts heating the trap and injects the analytes after the predefined desorption temperature has been reached. The second (Vol_inj) aspirates a fraction of the defined injection volume (50 % by default) and then starts to heat the trap, while simultaneously aspirating the remaining fraction of the injection volume. Analyte injection is performed solely, when the desorption temperature and the whole injection volume have been reached. In this way, the analytes will be transported into the syringe at first and then be injected through the heated bed with a higher injection flow, similar to a classical headspace injection technique. This is used to avoid peak broadening of volatile compounds and to compensate for the thermal expansion of the gas in the trap, during the heating process, which would otherwise result in bleeding of analytes into the injection system, before the actual injection is performed.

**Fig. 7:**
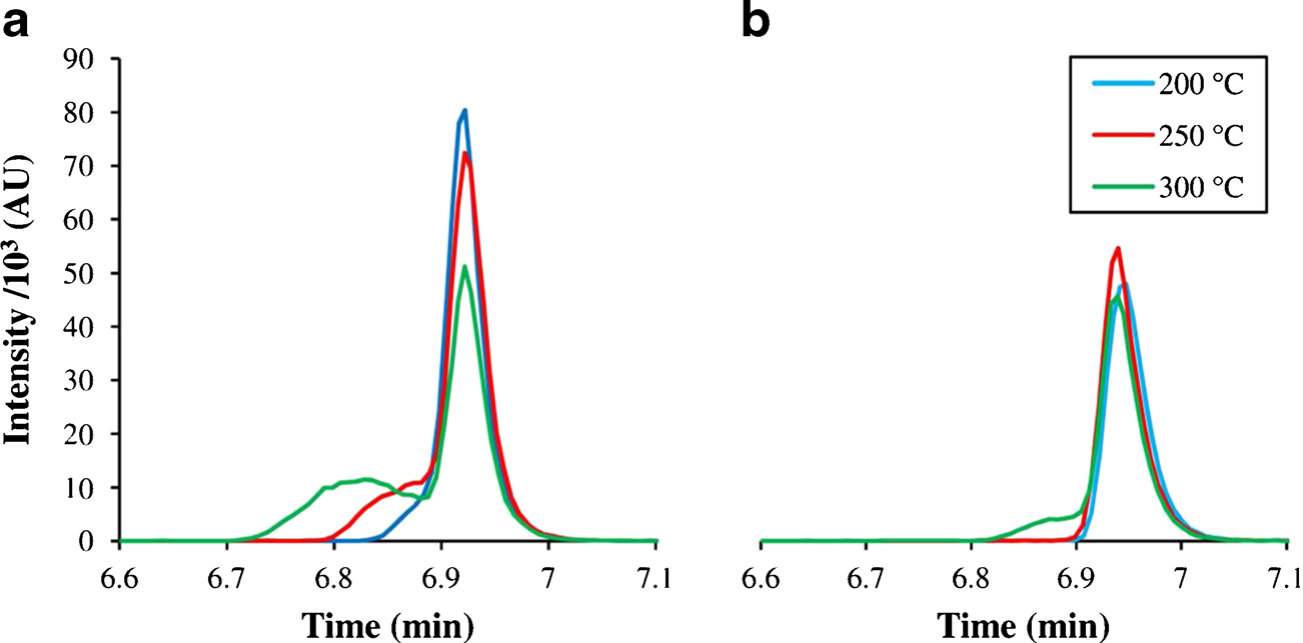
Peak shapes of 0.1 mg L^**−**1^ toluene from a Tenax TA trap using the (**a**) ITEX_inj and (**b**) Vol_inj methods at different desorption temperatures, with an injection volume of 500 μL, a desorption flow of 100 μL s^**−**1^, and a split ratio of 1:10

The differences between both injection methods for desorption temperatures of 200, 250, and 300 °C are presented in Fig. 7. The peaks with the ITEX_inj method displayed increased fronting, when the desorption temperature was raised, until a distinct valley developed at 300 °C. While the peak areas were similar with 204,794, 221,662, and 201,930 AU, respectively, the intensity and signal-to-noise ratio decreased significantly. The peaks of the Vol_inj method did not show fronting, except for the one with 300 °C desorption temperature, where the heating took longer than the parallel aspiration of the desorption gas volume, which might be compensated by a larger desorption volume. However, the peak areas were much smaller with 133,017, 139,258, and 135,774 AU, because the analytes were diluted to the whole extraction volume plus the void volume of the trap, which remains in the tube after the injection. The total volume of an ITEX tube is about 300 μL, and the sorbent bed takes up 160 μL, which results in about 118 μL of the sorbent material, if an optimal sphere packing is assumed, ensuing a total void volume of about 180 to 190 μL. With a desorption volume of 500 μL, the resulting peak area of the Vol_inj method should theoretically be around 40 % lower than the ITEX_inj peak area, which is close to the actual results.

The heating times for several desorption temperatures, starting from a temperature of 30 °C, are given in ESM Table S2, together with the theoretical expansion of the gas in the void volume. However, the theoretical expansion can only be used as an allusion to the necessary aspiration volume of the Vol_inj method, because the volume of gas released during heating is also influenced by the amount of analytes or water sorbed to the trap material. Above 200 °C, the heating rate decreases significantly, which would require larger desorption volumes with the Vol_inj method to avoid premature bleeding of the analytes to the injector, as it was observed with the 300 °C desorption temperature. On the other hand, this would also lead to stronger analyte dilution in the desorption gas and a broadened injection band. The peak width is also determined by the quotient of desorption volume and desorption flow (like in the example 500 ÷ 100 μL s^−1^ = 5 s), but the influence of the desorption flow is mostly insignificant for volatile compounds, while low volatiles benefit from lower flows.

In the given example, complete desorption of the analyte was achieved at 200 °C with both injection methods using a desorption volume of 500 μL and the peak areas did not further increase, when the desorption temperature has been raised. In this case, the ITEX_inj method gave a higher peak area with sufficiently good peak shape and might be the better option, when only volatile compounds are analyzed. Higher desorption temperatures may be needed, when also low volatile compounds are analyzed, necessitating the use of the Vol_inj method. Then, the analyst has to find a suitable balance between desorption temperature, desorption volume, and aspiration flow for all target analytes.

#### Injection with analyte focusing

The initial width and shape of the injection band are of less importance, when the analytes can be re-focused on the column. Therefore, the ITEX_inj method can be used with higher desorption temperatures, which will result in larger peak areas of low volatile compounds or when stronger sorbent materials than Tenax TA in the example above are used. In this case, the desorption temperature is either limited by the thermal stability of the analytes and the sorbent material or by the maximum temperature of the ITEX heater. Variations of the desorption volume only cause small effects on the resulting peak areas, because a fraction of the analytes will be transported into the GC injector by the thermal expansion during the heating process and only the remaining void volume needs to be flushed. Generally, the results with 100 and 500 μL desorption volume were very similar and a further increase to 1 mL only resulted in inferior repeatability for most compounds.

**Fig. 8:**
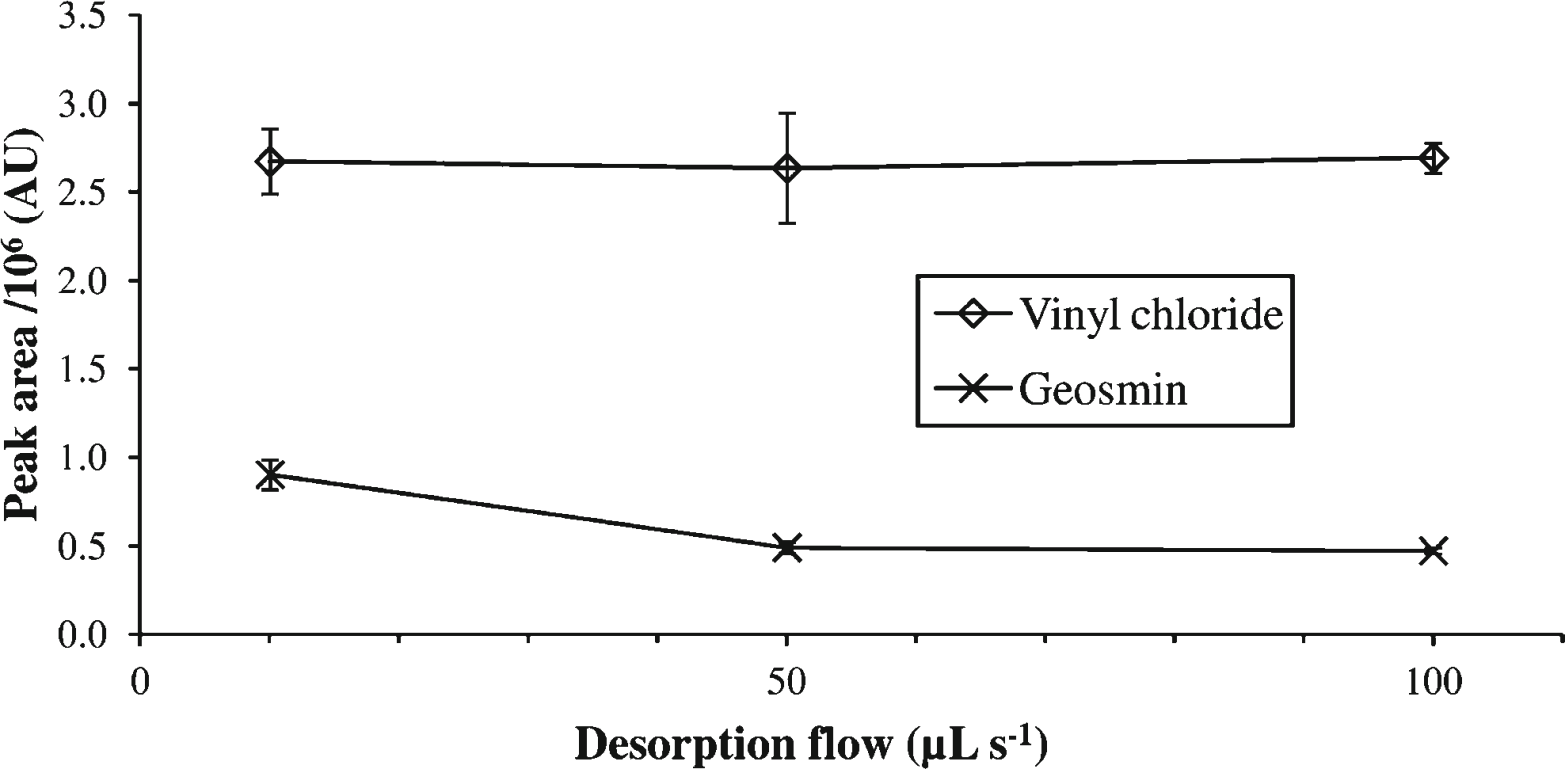
Influence of the desorption flow on the peak area of the very volatile vinyl chloride and the low volatile geosmin (taken from [4])

In contrast to the non-focused injection, low desorption flows do not result in peak broadening and, therefore, also low flows can be applied for injections when analyte focusing is possible. As mentioned before, the desorption flow had only low influence on the resulting peak areas of volatile compounds and was practically insignificant for highly volatiles like vinyl chloride, while a decrease of the desorption flow from 50 to 10 μL s^−1^ almost doubled the obtained peak area of geosmin (see Fig. 8).

### Trap conditioning

Before the first use, the traps should be conditioned to remove possible impurities from packing, transport, and storage. The conditioning is straightforward, the nitrogen flow is recommended to be about 5 mL min^−1^, and the conditioning temperature should be just below the maximum tolerable temperature of the sorbent material to achieve complete desorption of possible residual compounds. An initial conditioning time around 30 min should be sufficient. The conditioning time between analyses depends on the sorbent strength, the volatility of analytes, and the sample concentration. A flushing time of 10 min is usually long enough to avoid carryover of volatiles in the micrograms per liter range, while the complete removal of semi-volatiles in the milligrams per liter range can require over 20 min.

### Improving the ITEX procedure

**Fig. 9:**
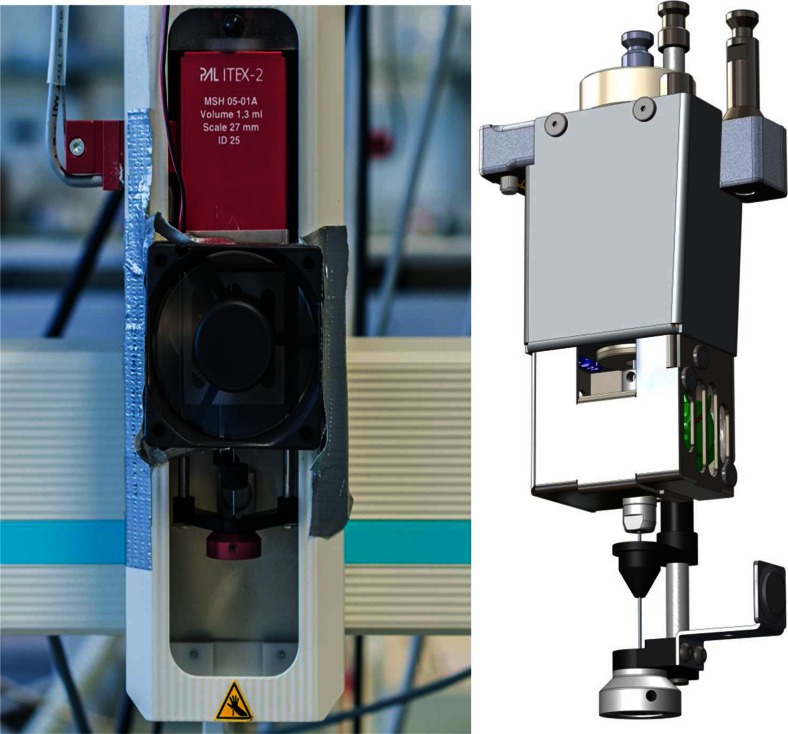
*Left* Combi PAL modified with a 6-cm fan for trap cooling. *Right* ITEX DHS with implemented fan as it is distributed by CTC Analytik GmbH (Zwingen, Switzerland). Scheme with permission by CTC Analytik GmbH

**Fig. 10:**
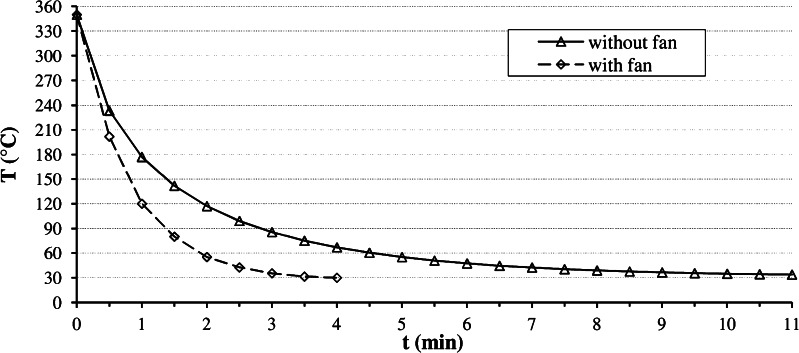
Influence of active cooling of the trap on cool-down time

A relatively simple way to improve the system is to add a cooling fan to the autosampler (Fig. 9) to reach lower trap temperatures, because this makes the sorption process more efficient. Therefore, a 12-V, 6-cm axial flow fan has been attached directly to the autosampler head using a duct tape. The fan was operated with a 5-V DC power adapter, originally intended for a USB hub, which gave sufficient air flow for the cooling task. A trap temperature of 24 °C could be achieved with active cooling, but the lowest software-controlled temperature of the ITEX trap heater, to maintain steady enrichment conditions, is 30 °C. This lower temperature limit could not be reached without active cooling, even in an air-conditioned laboratory. Active cooling also shortens the cooling time of the trap. Figure 10 shows the difference in cooling time between the standard passive cooling and the active cooling by a fan, attached to the autosampler. With a cooling fan, suitable trap temperatures for analyte enrichment can be reached in about one third of the time. CTC Analytik GmbH included a fan in the ITEX DHS device (see Fig. 9, right panel).

The most time-consuming steps in the ITEX procedure are typically sorption, sample conditioning, and trap cleaning with cooling. As seen before, the number of extraction strokes performed during the sorption step is the most important parameter, defining the sensitivity of the method, and it is therefore desirable to use as much time as possible on this step, when trace analysis is required. This can be achieved by the modification of the standard procedure to perform the trap cleaning in parallel to the sample conditioning, before extraction of the next sample begins (see ESM Fig. S7). When the whole extraction procedure is conducted in parallel to the GC run of the previous sample, it is possible to perform about 50 extraction strokes in a total analysis time of 30 min, with an incubation time of 15 min, while the trap is flushed for 10 min to allow a safe cooling time. The standard procedure would only allow 10 to 20 strokes, when the flushing time is shortened. Furthermore, the Vol_inj method by default aspirates 50 % of the desorption volume before heating the trap, which does not leave enough aspiration volume/time to achieve high desorption temperatures without analytes bleeding to the column, unless large desorption volumes are used. A modification to the macro that was performed without negative effects was to skip the pre-heating aspiration and to begin the aspiration of the whole desorption volume at the same time as the heating process. The aspiration flow can then be adjusted in a way that the required heating time (see ESM Table S2) is slightly shorter than the aspirating time.

### Possible sources of error

The main reasons for unsatisfactory results often are non-optimum extraction and injection conditions, like a small number of slowly performed extraction strokes or a too large desorption volume for volatile compounds. However, there are also a few mechanical issues that can cause the diminishing of extraction performance over time and need to be observed.

The most important is the plunger of the syringe. It should be checked for leak tightness regularly because a failure will result in less gas pumped through the sorbent bed, lowering the extracted analyte amount. Although the plunger usually lasts for several thousand movements, this limit might be reached within several weeks, when a very large number of extraction strokes are performed per analysis. Another fault can occur at the connection of the sorbent tube to the syringe. When the connection nut has not been tightened well enough, it can loosen over time due to thermal stress during desorption and trap cleaning that will result in a leakage, too. A problem that has only occurred once so far in our lab, in over 4 years of continuous use, was the blocking of the needle by scraped septum particles, which has most likely been caused by too much force on the septum nut.

## Conclusions and outlook

Based on previous experiences, the time needed for the development of appropriate ITEX-based methods for certain analytical tasks can be shortened drastically. Therefore, all characteristics of the sample and the analytes have to be taken into account. ESM Fig. S8 presents a flow chart in which the previously discussed parameters are summarized and which gives recommendations for efficient method development, for different analyte volatilities and sample compositions. This should enable new ITEX users to develop suitable methods in less time, avoiding unfavorable extraction and injection conditions. In an ongoing approach, a characterization of ITEX traps is undergoing with several test compounds to build a database, which can be used to predict optimal extraction conditions by simulation.

## Electronic supplementary material

ESM 1 (PDF 800 kb)

## References

[1] Grote C, Levsen K, Pawliszyn J (1999) The application of SPME in water analysis. In: Applications of solid phase microextraction. The Royal Society of Chemistry, pp. 169-187. doi: 10.1039/9781847550149-00169

[2] Musshoff F, Lachenmeier DW, Kroener L, Madea B (2002). Automated headspace solid-phase dynamic extraction for the determination of amphetamines and synthetic designer drugs in hair samples. J Chromatogr A.

[3] Jochmann MA, Yuan X, Schilling B, Schmidt TC (2008). In-tube extraction for enrichment of volatile organic hydrocarbons from aqueous samples. J Chromatogr A.

[4] Laaks J, Jochmann MA, Schilling B, Schmidt TC (2010). In-tube extraction of volatile organic compounds from aqueous samples: an economical alternative to purge and trap enrichment. Anal Chem.

[5] Kolb B, Ettre LS (2006) Sample handling in HS-GC. In: Static headspace–gas chromatography. Wiley, pp. 165-196. doi: 10.1002/0471914584.ch4

[6] Rudling J (1990). Improvement of activated carbon for air sampling. J Chromatogr A.

[7] Dettmer K, Engewald W (2002). Adsorbent materials commonly used in air analysis for adsorptive enrichment and thermal desorption of volatile organic compounds. Anal Bioanal Chem.

[8] Dettmer K, Knobloch T, Engewald W (2000). Stability of reactive low boiling hydrocarbons on carbon based adsorbents typically used for adsorptive enrichment and thermal desorption. Fresenius J Anal Chem.

[9] Kornacki W, Fastyn P, Gierczak T, Gawlowski J, Niedzielski J (2006). Reactivity of carbon adsorbents used to determine volatile organic compounds in atmospheric air. Chromatographia.

[10] Slabizki P, Potouridis TH-GS (2014). On trap mass spectrometric effect leading to peak suppression and solvent-free peak focusing due to co-chromatography. Chromatographia.

[11] Cao X-L, Hewitt CN (1994). Study of the degradation by ozone of adsorbents and of hydrocarbons adsorbed during the passive sampling of air. Environ Sci Technol.

[12] Seethapathy S, Gorecki T (2012). Applications of polydimethylsiloxane in analytical chemistry: a review. Anal Chim Acta.

[13] van Pinxteren M, Paschke A, Popp P (2010). Silicone rod and silicone tube sorptive extraction. J Chromatogr A.

[14] Pawliszyn J (2003). Sample preparation: Quo Vadis?. Anal Chem.

[15] Zapata J, Mateo-Vivaracho L, Lopez R, Ferreira V (2012). Automated and quantitative headspace in-tube extraction for the accurate determination of highly volatile compounds from wines and beers. J Chromatogr A.

[16] Schwarzenbach RP, Gschwend PM, Imboden DM (2005) Partitioning: molecular interactions and thermodynamics. In: Environmental organic chemistry. Wiley, pp. 57-96. doi: 10.1002/0471649643.ch3

[17] Schwarzenbach RP, Gschwend PM, Imboden DM (2005) Sorption I: general introduction and sorption processes involving organic matter. In: Environmental organic chemistry. Wiley, pp. 275-330. doi: 10.1002/0471649643.ch9

[18] Zapata J, Lopez R, Herrero P, Ferreira V (2012). Multiple automated headspace in-tube extraction for the accurate analysis of relevant wine aroma compounds and for the estimation of their relative liquid-gas transfer rates. J Chromatogr A.

[19] Niu L, Bao J, Zhao L, Zhang Y (2011). Odor properties and volatile compounds analysis of Torreya grandis aril extracts. J Essent Oil Res.

[20] Rasanen I, Viinamäki J, Vuori E, Ojanperä I (2010). Headspace in-tube extraction gas chromatography-mass spectrometry for the analysis of hydroxylic methyl-derivatized and volatile organic compounds in blood and urine. J Anal Toxicol.

[21] Hüffer T, Osorio XL, Jochmann MA, Schilling B, Schmidt TC (2013). Multi-walled carbon nanotubes as sorptive material for solventless in-tube microextraction (ITEX2)—a factorial design study. Anal Bioanal Chem.

[22] van Deemter JJ, Zuiderweg FJ, Klinkenberg A (1995). Longitudinal diffusion and resistance to mass transfer as causes of nonideality in chromatography. Chem Eng Sci.

[23] Lord HL, Zhan W, Pawliszyn J (2010). Fundamentals and applications of needle trap devices: a critical review. Anal Chim Acta.

[24] Wang A, Fang F, Pawliszyn J (2005). Sampling and determination of volatile organic compounds with needle trap devices. J Chromatogr A.

