# Supplementary material for: Optimization strategies of in-tube extraction (ITEX) methods
Source: Anal Bioanal Chem. 2015 Jun 30;407(22):6827–38. doi: 10.1007/s00216-015-8854-4 (PMC4545181; doi:10.1007/s00216-015-8854-4)
Supplement: Supplementary file 1 — (PDF 800 kb) [file 216_2015_8854_MOESM1_ESM.pdf]

Analytical and Bioanalytical Chemistry

Electronic Supplementary Material

Optimization strategies of in-tube extraction (ITEX) methods

Jens Laaks, Maik A. Jochmann, Beat Schilling, Torsten C. Schmidt

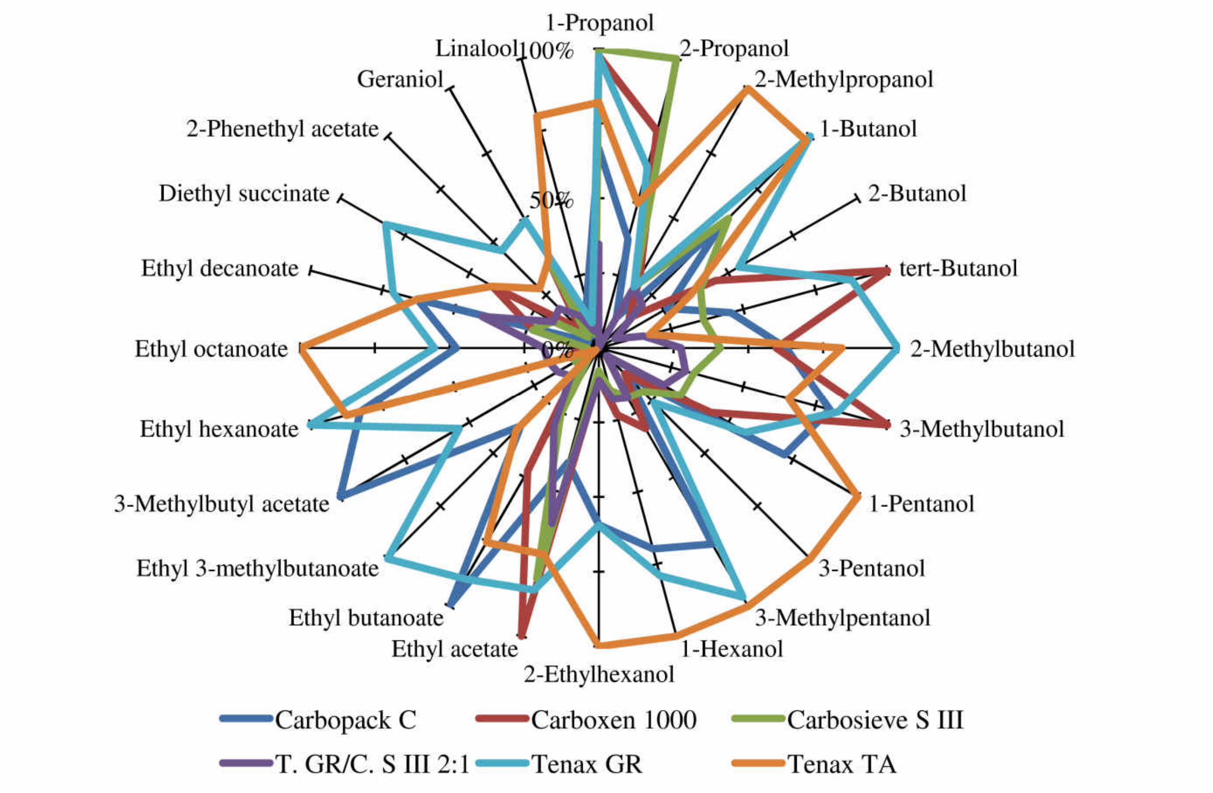

Fig. S1 Relative extraction yields of six tested standard sorbent traps for the analysis of beer aroma compounds, result for each compound was normalized to the most efficient sorbent from Fig. S1 and Fig. S2

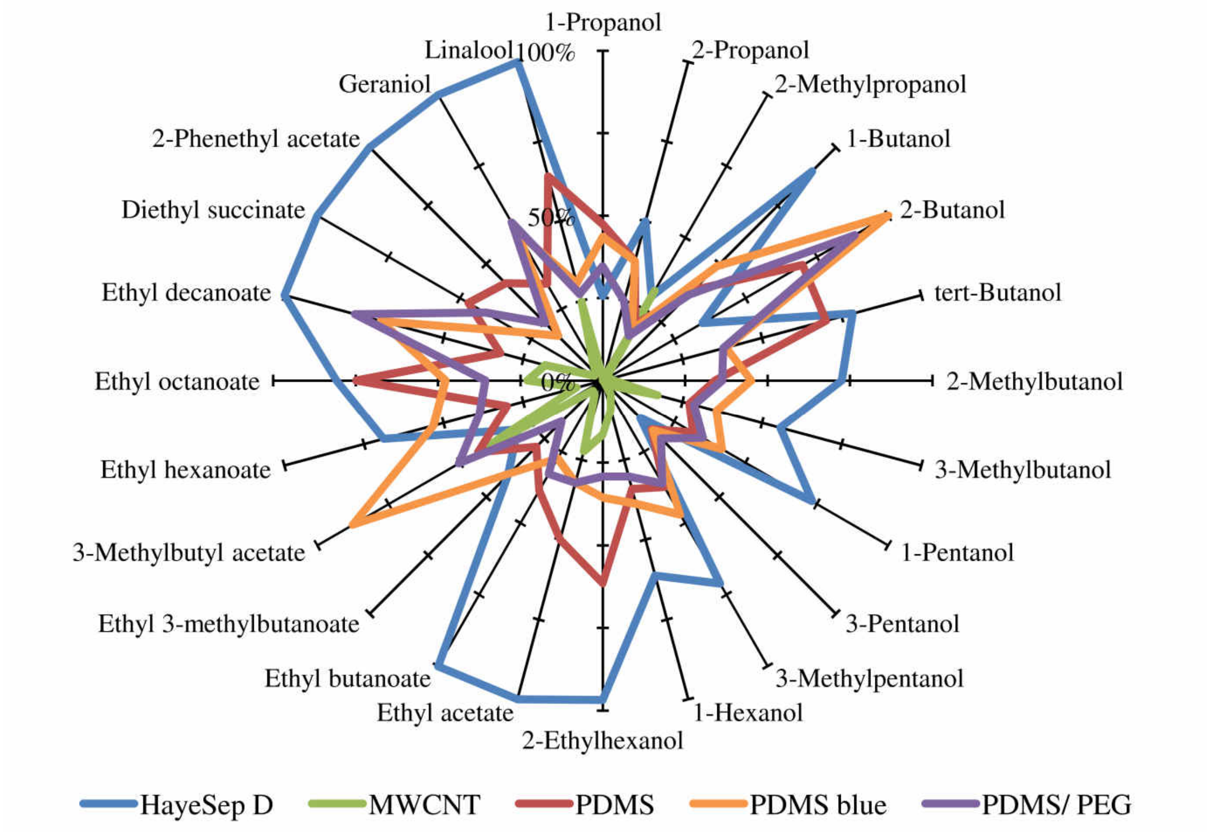

Fig. S2 Relative extraction yields of five tested custom packed traps for the analysis of beer aroma compounds, result for each compound was normalized to the most efficient sorbent from Fig. S1 and Fig. S2

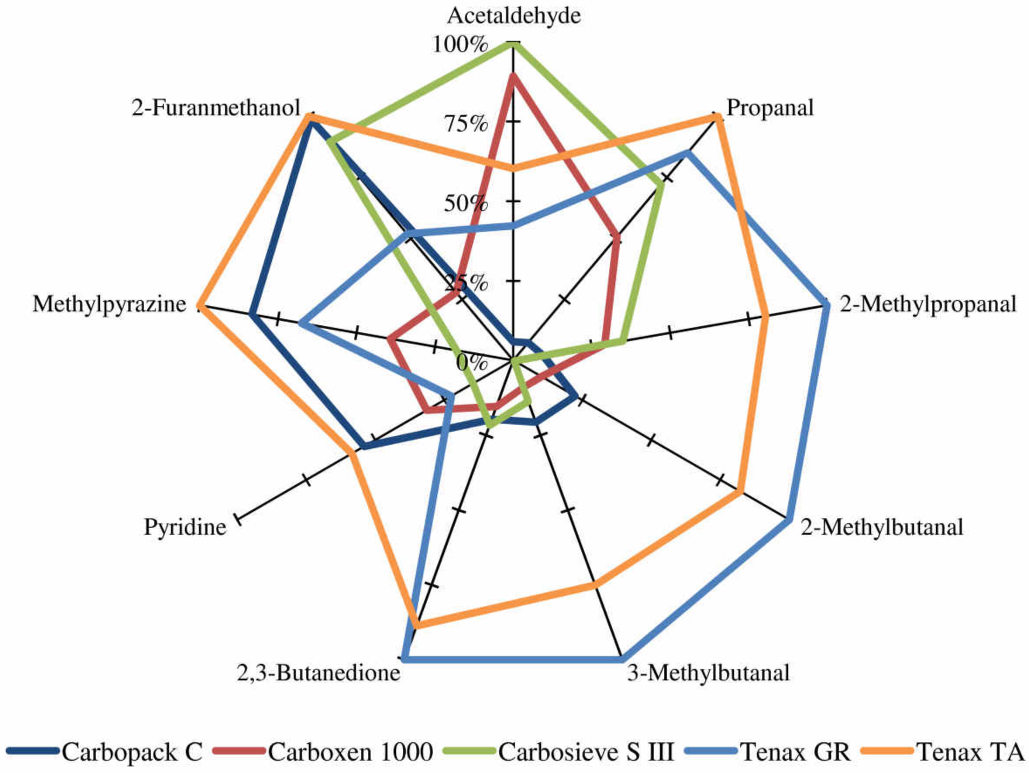

Fig. S3 Relative extraction yields of five tested standard sorbent traps for the analysis of coffee aroma compounds, result for each compound was normalized to the most efficient sorbent from Fig. S3 and Fig. S4

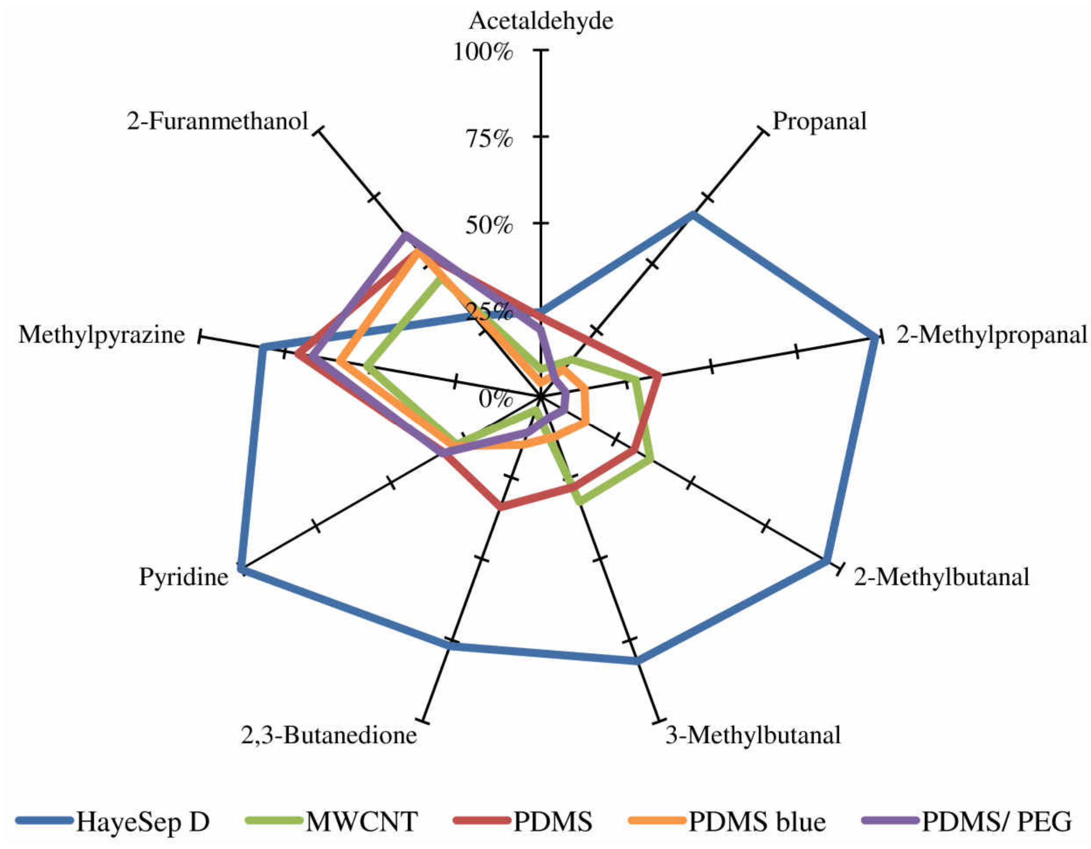

Fig. S4 Relative extraction yields of five tested custom packed traps for the analysis of coffee aroma compounds, result for each compound was normalized to the most efficient sorbent from Fig. S3 and Fig. S4

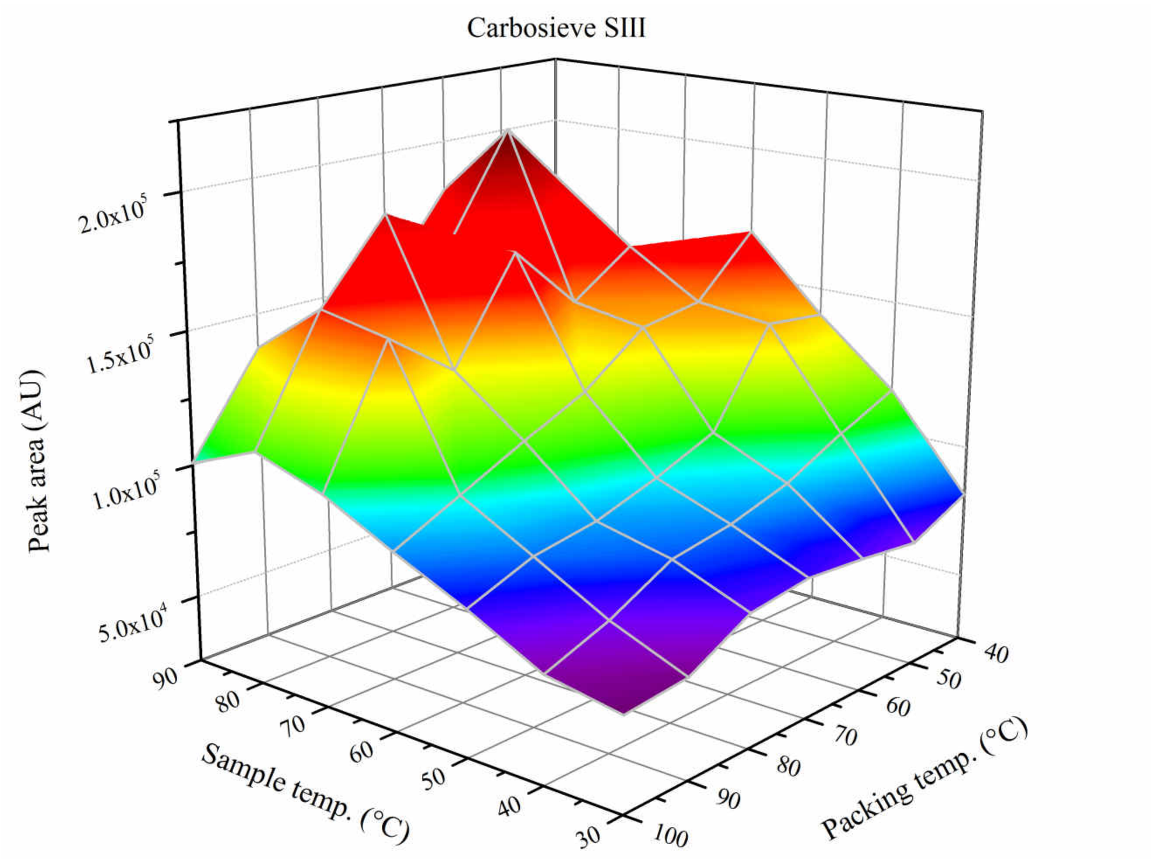

Fig. S5 Influence of sample and packing temperature on the resulting peak area of toluene for Carbosieve S III

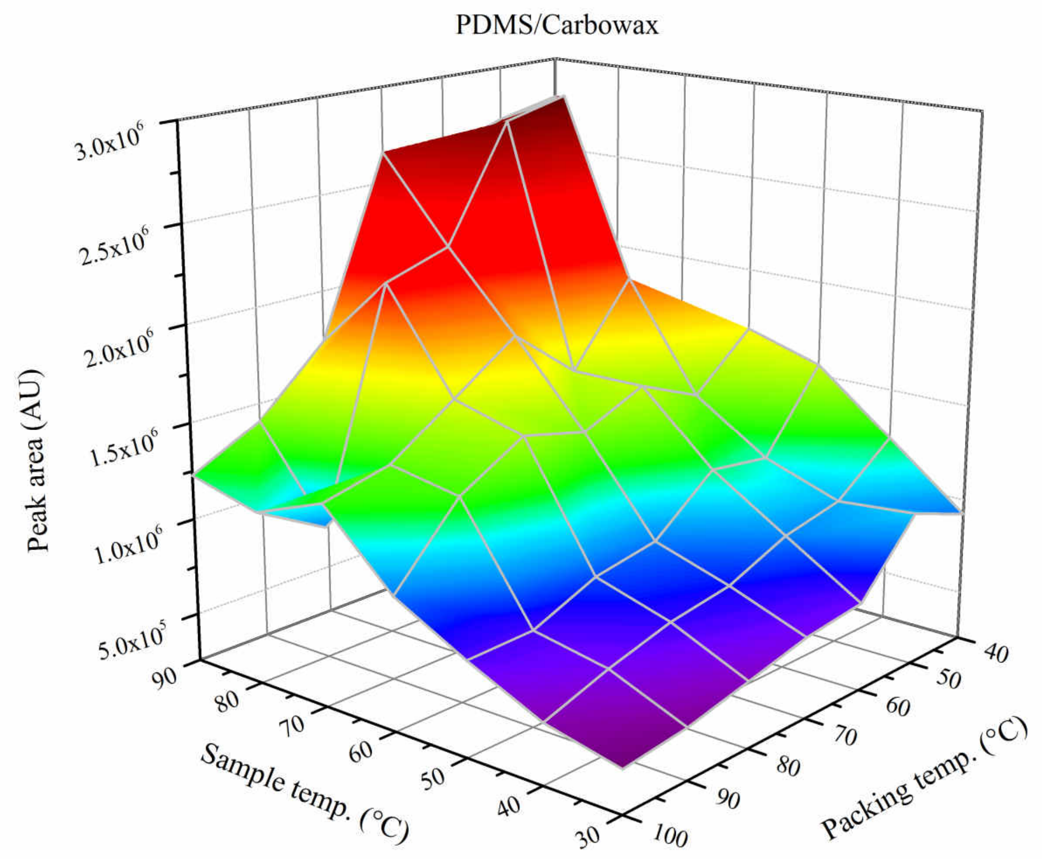

Fig. S6 Influence of sample and packing temperature on the resulting peak area of toluene for PDMS with 10% Carbowax

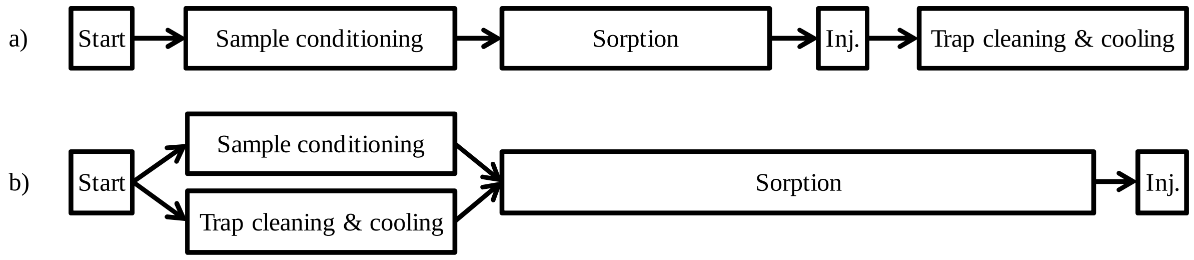

Fig. S7 Basic steps of the ITEX-procedure; a) standard procedure, b) runtime optimized procedure

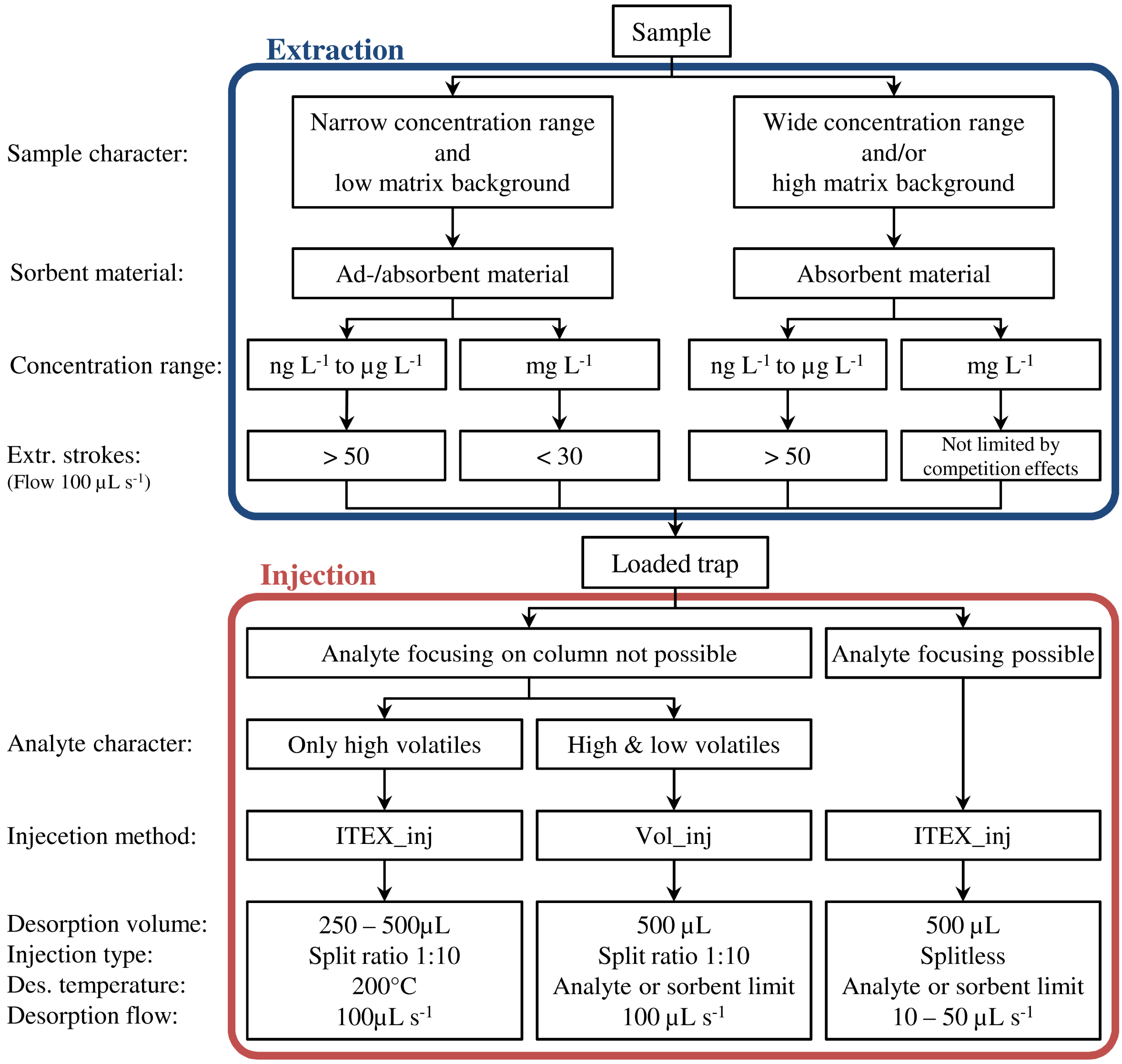

Fig. S8 Flow chart of the ITEX procedure for accelerated method development, showing exemplary extraction and injection parameters for different sample conditions and analyte compositions

Table S1 Extraction efficiencies of nine combinations of numbers of extration strokes and extraction flow, calculated as resulting peak area per second of the extraction procedure

**Extraction strokes (n)**	20	50	80
**Extraction flow (µL s^{-1})**	**Extraction efficiency (kAU s^{-1})**		
30	4.9	2.2	1.7
60	6.1	3.6	2.6
90	6.7	5.0	3.4

Table S2 Heating time and theoretical void gas expansion during the desorption process, starting from 30 °C

**T (°C)**	30	50	100	150	200	250	300	350
**Heating time (s)**	0	5	10	16	24	34	47	63
**Volume expansion (%)**	0	7	23	40	56	73	89	106
